# Supplementary material for: The effect of circular stapler size on anastomotic stricture formation in colorectal surgery: A propensity score matched study
Source: PLoS One. 2023 Oct 30;18(10):e0287595. doi: 10.1371/journal.pone.0287595 (PMC10615279; doi:10.1371/journal.pone.0287595)
Supplement: S1 File — (DOCX) [file pone.0287595.s001.docx]

Supplement Table 1. Clinical characteristics between two groups in colorectal anastomosis

| Variables | Colorectal anastomosis | | | |
| --- | --- | --- | --- | --- |
|  | 28/29mm | | 25mm | p |
|  | N=57 | | N=48 |  |
| Age (years) | 61.4 ± 12.4 | | 64.9 ± 12.2 | 0.145 |
| Sex |  | |  | 0.845 |
| Male | 29 (50.9%) | | 26 (54.2%) |  |
| Female | 28 (49.1%) | | 22 (45.8%) |  |
| Height (cm) | 157.6 ± 9.4 | | 159.0 ± 9.5 | 0.468 |
| Weight (kg) | 60.6 ± 11.4 | | 60.4 ± 11.6 | 0.939 |
| Body mass index (kg/m^2^) | 24.7 ± 4.9 | | 24.0 ± 3.9 | 0.443 |
| Diabetes | 13 (22.8%) | | 12 (25.0%) | 0.822 |
| Hypertension | 26 (45.6%) | | 26 (54.2%) | 0.436 |
| Heart disease | 6 (10.5%) | | 4 (8.3%) | 0.751 |
| Pulmonary disease | 3 (5.3%) | | 5 (10.4%) | 0.465 |
| Liver disease | 1 (1.8%) | | 1 (2.1%) | >0.999 |
| Cerebrovascular disease | 4 (7.0%) | | 3 (6.3%) | >0.999 |
| ASA classification |  | |  | >0.999 |
| 1 | 8 (14.0%) | | 3 (6.3%) |  |
| 2 | 34 (59.6%) | | 35 (72.9%) |  |
| 3 | 13 (22.8%) | | 9 (18.8%) |  |
| 4 | 2 (3.5%) | | 1 (2.1%) |  |
| Smoking history |  | |  | 0.863 |
| None | 39 (68.4%) | | 35 (72.9%) |  |
| Past | 7 (12.3%) | | 5 (10.4%) |  |
| Present | 11 (19.3%) | | 8 (16.7%) |  |
| Alcoholic history | 15 (26.3%) | | 13 (27.1%) | >0.999 |
| Emergency surgery | 11 (19.3%) | | 10 (20.8%) | >0.999 |
| Benign or cancer |  | |  | 0.552 |
| Benign | 22 (38.6%) | | 22 (45.8%) |  |
| Cancer | 35 (61.4%) | | 26 (54.2%) |  |
| Diversion |  | |  | 0.139 |
| None | 43 (75.4%) | | 29 (60.4%) |  |
| Yes | 14 (24.6%) | | 19 (39.6%) |  |
| Surgery time (min) | 239.1 ± 89.2 | | 210.2 ± 84.0 | 0.093 |
| Abbreviation : ASA, American Society of Anesthesiologists | |  |  |  |

Supplement Table 2. Anastomosis problems and Clavien-dindo Classification

|  | 28/29mm | 25mm | p-value |
| --- | --- | --- | --- |
| Anastomotic problem |  |  |  |
| Total | 5 (8.8%) | 2 (4.2%) | 0.450 |
| Stricture | 4 (7.0%) | 2 (4.2%) | 0.686 |
| Leakage | 3 (5.3%) | 0 | 0.248 |
| Bleeding | 0 | 0 | >0.999 |
| Clavien-Dindo Classification |  |  | 0.166 |
| 0 | 32 (56.1%) | 31 (64.6%) |  |
| 1 | 9 (15.8%) | 6 (12.5%) |  |
| 2 | 10 (17.5%) | 11 (22.9%) |  |
| 3a | 3 (5.3%) | 0 |  |
| 3b | 3 (5.3%) | 0 |  |
|  |  |  |  |

Supplement Table 3. Univariate and multivariate analysis for factors affecting an anastomotic stricture

|  |  | Stricture | | Univariate analysis | | | Multivariate analysis | | |
| --- | --- | --- | --- | --- | --- | --- | --- | --- | --- |
|  |  | None | Present | OR | 95% CI | p-value | Adjusted OR | 95% CI | p-value |
| Stapler size | 28/29 mm | 53 (93.0%) | 4 (7.0%) | 0.58 | 0.10-3.29 | 0.686 |  |  |  |
|  | 25mm | 46 (95.8%) | 2 (4.2%) |  |  |  |  |  |  |
| Anastomotic leakage | None | 98 (96.1%) | 4 (3.9%) | 49.00 | 3.64 - 659.95 | **0.008** | 39.38 | 2.49 - 623.37 | **0.009** |
|  | Present | 1 (33.3%) | 2 (66.7%) |  |  |  |  |  |  |
| Age (years) | < 70 | 64 (94.1%) | 4 (5.9%) | 0.91 | 0.16 - 5.24 | >0.999 |  |  |  |
|  | ≧70 | 35 (94.6%) | 2 (5.4%) |  |  |  |  |  |  |
| Sex | Male | 52 (94.5%) | 3 (5.5%) | 1.11 | 0.21- 5.75 | >0.999 |  |  |  |
|  | Female | 47 (94.0%) | 3 (6.0%) |  |  |  |  |  |  |
| Body mass index (kg/m^2^) | < 25 | 62 (93.9%) | 4 (6.1%) | 0.84 | 0.15 - 4.80 | >0.999 |  |  |  |
|  | ≧25 | 37 (94.9%) | 2 (5.1%) |  |  |  |  |  |  |
| Diabetes | None | 75 (93.8%) | 5 (6.2%) | 0.63 | 0.07 – 5.62 | >0.999 |  |  |  |
|  | Present | 24 (96.0%) | 1 (4.0%) |  |  |  |  |  |  |
| Hypertension | None | 48 (90.6%) | 5 (9.4%) | 0.19 | 0.02 – 1.67 | 0.205 |  |  |  |
|  | Present | 51 (98.1%) | 1 (1.9%) |  |  |  |  |  |  |
| Heart disease | None | 89 (93.7%) | 6 (6.3%) | 0.94 | 0.89 - 0.99 | >0.999 |  |  |  |
|  | Present | 10 (100%) | 0 |  |  |  |  |  |  |
| Pulmonary disease | None | 92 (94.8%) | 5 (5.2%) | 2.63 | 0.27 – 25.71 | 0.386 |  |  |  |
|  | Present | 7 (87.5%) | 1 (12.5%) |  |  |  |  |  |  |
| Liver disease | None | 97 (94.2%) | 6 (5.8%) | 0.94 | 0.90 - 0.99 | >0.999 |  |  |  |
|  | Present | 2 (100%) | 0 |  |  |  |  |  |  |
| Cerebrovascular disease | None | 94 (95.9%) | 4 (4.1%) | 9.4 | 1.38- 64.18 | 0.051 | 7.22 | 0.76 – 68.58 | 0.085 |
|  | Present | 5 (71.4%) | 2 (28.6%) |  |  |  |  |  |  |
| ASA classification | 1,2 | 75 (93.8%) | 5 (6.2%) | 0.63 | 0.07 – 5.62 | >0.999 |  |  |  |
|  | 3,4 | 24 (96.0%) | 1 (4.0%) |  |  |  |  |  |  |
| Smoking history | None | 70 (94.6%) | 4 (5.4%) | 1.21 | 0.21- 6.96 | >0.999 |  |  |  |
|  | Present | 29 (93.5%) | 2 (6.5%) |  |  |  |  |  |  |
| Alcholic history | None | 73 (94.8%) | 4 (5.2%) | 1.40 | 0.24 - 8.12 | 0.656 |  |  |  |
|  | Present | 26 (92.9%) | 2 (7.1%) |  |  |  |  |  |  |
| Emegency or elective | Elective | 78 (92.9%) | 6 (7.1%) | 0.93 | 0.88 - 0.99 | 0.597 |  |  |  |
|  | Emengency | 21 (100%) | 0 |  |  |  |  |  |  |
| Benign or cancer | Benign | 42 (95.5%) | 2 (4.5%) | 1.47 | 0.26 – 8.43 | >0.999 |  |  |  |
|  | Cancer | 57 (93.4%) | 4 (6.6%) |  |  |  |  |  |  |
| Diversion | None | 68 (94.4%) | 4 (5.6%) | 1.10 | 0.20 – 6.31 | >0.999 |  |  |  |
|  | Present | 31 (93.9%) | 2 (6.1%) |  |  |  |  |  |  |
| Abbreviation : ASA, American Society of Anesthesiologists ; OR, Odd ratios ; CI, confidence interval | | | | | |  |  |  |  |
