# Supplementary material for: The effect of circular stapler size on anastomotic stricture formation in colorectal surgery: A propensity score matched study
Source: PLoS One. 2023 Oct 30;18(10):e0287595. doi: 10.1371/journal.pone.0287595 (PMC10615279; doi:10.1371/journal.pone.0287595)
Supplement: Attachment — Submitted filename: Response_to_Reviewers.docx [file pone.0287595.s002.docx]

**Editor Comments:**

I believe that the reviewers have focused on the weaknesses of the paper, while also providing insights to correct them.

I think some of the reviewers' considerations play a crucial role in meaningfully improving the paper:

“….Since the risk profile for anastomotic stenosis and also anastomotic leaks differs significantly from ileocolic anastomoses and colorectal anastomoses, I consider the selection of the patient collective to be unsuitable. As confirmed in your data, only an evaluation of the colorectal anastomoses makes sense.

… I suggest to include only those patients who underwent left colectomy or anterior resection, ….In addition, the impact of a derivative stoma should be taken in consideration during the analysis of the results.

…. please could the Authors specify why the circular stapler sized > 29-mm (widely used in rectal cancer surgery) have been excluded.

… Who and why chooses a 25-mm or 28/29-mm stapler?”

An improvement in English would be desirable.


Reviewers' comments:

**Reviewer #1: The authors report the effect of cicular stapler size on anastomotic stricture in colorectal surgery.**

This is a relevant question in colorectal surgery and therefore an interesting topic. Overall, the conduct of the study is correct and the manuscript is in order. An improvement in English would be desirable.

However, I have one major concern:
Since the risk profile for anastomotic stenosis and also anastomotic leaks differs significantly from ileocolic anastomoses and colorectal anastomoses, I consider the selection of the patient collective to be unsuitable. As confirmed in your data, only an evaluation of the colorectal anastomoses makes sense.

**Answer>**

We thank the reviewer for their comments.

As per the reviewer’s recommendation, we performed a subgroup analysis for 105 patients with colorectal anastomosis. Even in this subgroup analysis, there was no difference in anastomotic stricture between the two groups.

We described the relevant procedure in the revised manuscript (line 170) and included the data in supplemental tables (S1-S3).

We revised the manuscript to improve the language.

**Reviewer #2: Thanks to the Authors for this interesting paper.**

I would like to make the following considerations and questions.


METHODS

Patients

The Authors assert that the purpose of the study is to determine whether the frequency of anastomotic stricture depends on the stapler size: please could the Authors specify why the circular stapler sized > 29-mm (widely used in rectal cancer surgery) have been excluded.

**Answer>**

We appreciate your considerate comments. In our study data, only 12 (1.3%) out of a total of 894 patients used 29 mm or more. Additionally, in a study with a similar subject to ours^1,2^, a single group of 28/29-mm was used. Therefore, the group of “29 mm or more” was excluded. The number of patients corresponding to each stapler size in the excluded patient group has been added to Fig 1.

Refereces>

1. T. Nagaoka et al. Dis Colon Rectum. 2021 Aug 1;64(8):937-945.

2. T. Reif de Paula et al. Tech Coloproctol. 2020 Apr;24(4):283-290.

Procedure

Do the Authors usually or always perform end-to-side anastomosis by mean a circular stapler following ileocecal resection or right colectomy? Is a linear stapler used for section or closure of any bowel stump?
Who and why chooses a 25-mm or 28/29-mm stapler?

**Answer>**

At our institution, end-to-side anastomosis is always performed when performing ileocecal resection or right hemicolectomy, and a linear stapler is used for stump closure.

25-mm or 28/29-mm staplers are used entirely according to the surgeon's decision; therefore, the exact reason for this selection is unknown. We have added the relevant explanations to the procedure (line 85) and limitation sections (line 253) of the revised manuscript.

Statistical analyses

Please could the Authors specify the methodology for after matching analysis, for example McNemar’s test, paired t test or Wilcoxon signed rank test.

**Answer>**

After propensity score matching, the clinical characteristics were analyzed using the Mann–Whitney U test or Student’s t-test for continuous variables and the chi-squared test, Fisher’s exact test, or linear-by-linear association for categorical variables. A series of analyses related to propensity score matching was conducted using Web-based Analysis with R (prepared by web-r.org) based on the R package MatchIt. We have added this description to the methods section (line 111).

RESULTS

Baseline characteristics

In table 1 the clinical characteristics of patients are similar but 25-mm staplers are significantly more used in benign lesions (28.3% vs 8.1% of 28/29-mm stapler group) and emergency surgery (15.1% vs 4.8% of 28/29-mm stapler group) whilst 25-mm staplers are significantly less used in colorectal anastomosis (1 out of 23 – 4.3% – left colectomy patients and 47 out of 654 – 7.2% – anterior resection patients): how do the Authors motivate these so impressive differences?

**Answer>**

The reason for the occurrence of this discrepancy is unknown, as the size of the round stapler used was entirely at the discretion of the surgeon. However, to compensate for this difference, we performed a propensity score matching.

Outcomes

Anastomotic stenosis occurred in 6 cases, 4 in the 28/29-mm group (in 2 cases after anastomotic leak) and 2 in the 25-mm group (without any leak) and in table 3 only anastomotic leak resulted as independent risk factor for stricture at multivariate analysis: despite the small number of cases, is there any reason for stricture in patients without leak, especially in the 2 patients in the 25-mm group?

**Answer>**

Due to the small number of patients included in our study, determining the reason for stricture occurrence in the 25-mm group without the risk factors from our study was not possible. However, considering that stricture occurred in two patients in the 28/29-mm group without leakage, we believe that there was a reason other than the stapler size. Additional research is needed to elucidate this point; therefore, we have included it in the limitations section (line 255).

Anastomotic stricture management

Please could the Authors specify which stapler was used in the 3 patients that underwent operative treatment for symptomatic stricture.
Was the decision of stent insertion in the patient with stenosis after left colectomy dictated by the specific oncological stage of the disease or is stent insertion part of the armamentarium of strictures’ treatment?
How many finger dilatations were performed in order to obtain a permanently wide stricture site? Was pneumatic dilatation considered?

Please could the Authors provide details concerning follow up duration and long-term efficacy of treatment.

**Answer>**
In one patient using a 28-mm circular stapler, stricture was confirmed 95 days after surgery, and there was no further stricture after 2485 days with a single finger dilatation.

In one patient who used a 25-mm circular stapler, stricture was confirmed 157 days after surgery, but there was no further stricture for 385 days after one finger dilatation.

Patients who underwent left hemicolectomy using a 28-mm circular stapler developed anastomotic stricture after 60 days, and a stent was inserted to resolve the stricture. After stent insertion, symptoms improved, and the patients were discharged; however, follow-up was lost thereafter. When the medical records were checked, pneumatic dilatation was not considered for this patient.

We have added these details to the revised manuscript (line 186).

DISCUSSION

The Authors report that the 2011 Cochrane Database Systematic Review (reference 1) showed no differences in incidence of stricture following stapled anastomosis, compared with hand-sewn anastomosis, and correctly underline the small number of patients (65) included in the two studies evaluating the outcome “anastomotic stricture” (Izbicki JR et al. Der Chirurg 1998;69:725-34, published data only; Ikeuchi H et al. Digestive Surgery 2000;17:493-496, published and unpublished data); however, all the 26 “stapled” patients included in the two above mentioned studies underwent a side-to-side ileocolic anastomosis by mean of a linear and not circular stapler, therefore these data don't seem to have a relationship with the aim of the study.

**Answer>**

As per the reviewer’s comment, we have deleted the relevant section.

**Reviewer #3**: This is a manuscript that aims at evaluating the effect of circular stapler size on anastomotic stricture formation in colorectal surgery. The authors found no significant differences between the two groups. However, the interpretation of the results is biased by the small number of patients included in the two groups (the sample size calculation is not mentioned) and by the inclusion of both right and left colectomies/anterior resections. I suggest to include only those patients who underwent left colectomy or anterior resection, since the incidence of anastomotic stricture is negligible after right colectomy. In addition, the impact of a derivative stoma should be taken in consideration during the analysis of the results.
Lastly, the timing of anastomotic check is not clearly reported, as also the clinical relevance of the strictures (did the patients with stricture report any symptom? From the paragraph assessing the stenosis management, it seems the the clinical impact was minimal.

**Answer>**

We thank the reviewer for their considerate comments.

Following the reviewer’s recommendation, we performed a subgroup analysis for 105 patients with colorectal anastomosis and evaluated the effect of diversion (stoma) on anastomotic stricture (added: line 170; Table S1, S3).

Finally, we confirmed anastomotic stricture with colonoscopy for all patients (added: line 89).
